# PD-L1 correlates with chemokines and cytokines in gingival crevicular fluid from healthy and diseased sites in subjects with periodontitis

**DOI:** 10.1186/s13104-020-05376-9

**Published:** 2020-11-13

**Authors:** Andrew Shelby, Chandler Pendleton, Emma Thayer, Georgia K. Johnson, Xian Jin Xie, Kim A. Brogden

**Affiliations:** 1 Department of Periodontics, College of Dentistry, University of Iowa, Iowa City, IA USA; 2 Division of Biostatistics and Computational Biology, College of Dentistry, University of Iowa, Iowa City, IA USA; 3 Iowa Institute for Oral Health Research, College of Dentistry, University of Iowa, Iowa City, IA USA

**Keywords:** PD-1, PD-L1, Cytokine, Periodontal disease, Periodontitis

## Abstract

**Objective:**

PD-L1 is an immune checkpoint molecule that regulates immune and inflammatory responses. While cells of periodontal tissues express PD-L1, its presence in GCF is not known. The purpose of this study was to measure the PD-L1 values in GCF and correlate values with the presence of chemokine and cytokine values from periodontally diseased subjects and periodontally healthy subjects.

**Results:**

PD-L1 values (pg/30 s), determined in triplicate using a fluorescent microparticle-based immunoassay ranged from 0.04–31.65 pg/30 s. PD-L1 correlated with 15 out of 22 chemokine and cytokine responses. In 85 healthy sites in 31 subjects, PD-L1 values were negatively correlated with IL6, CXCL8, IL10, and CCL3 values. In 53 diseased sites in 20 subjects, PD-L1 values were positively correlated with CCL11, CSF2, IFNG, IL1A, IL1B, IL2, IL7, IL15, and CCL5 values and negatively correlated with IL12A and IL5 values. Gene ontology (GO) annotations identified roles of PD-L1 in Th1 and Th2 activation and T-cell exhaustion signaling canonical pathways. PD-L1 values were correlated with the expression of chemokines and cytokines, which likely regulates immune cell trafficking and protects the periodontium from uncontrolled immune responses to pathogens and inflammation-induced tissue damage.

## Introduction

PD-L1 is a 33.28 kDa type I transmembrane protein expressed on the surface of immune and non-immune cells [1–3]. It is a co-inhibitory and immune checkpoint protein that binds to receptor PD-1 on T-cells [4]. The PD-L1/PD-1 interaction regulates the balance between co-stimulatory and co-inhibitory immune signals and maintains the breadth and magnitude of immune responses. Increased expression of PD-L1 inhibits T-cell proliferation, reduces T-cell survival, inhibits cytokine release, promotes T-cell apoptosis, and leads to T-cell exhaustion and immunosuppression [5, 6].

Although this mechanism is thought to occur in periodontal disease to protect against inflammation-induced tissue damage [7], there are no known reports of PD-L1 detected in GCF or no known correlations of PD-L1 with the presence of chemokines and cytokines in GCF. Periodontal patients often demonstrate a reduced immune response that allows periodontopathogens to exert an exaggerated suppressive mechanism on T-cell function [8–15]. Demonstrating the presence of PD-L1 in GCF would support its role in protecting tissue against inflammation-induced damage by inhibiting T-cell proliferation and decreased cytokine production [15].

Tymkiw et al. reported that periodontally diseased subjects had significantly elevated cytokine and chemokine profiles [16]. The aim of our present study was to determine the concentrations of PD-L1 in the GCF of those same subjects and correlate their values with the presence of chemokines and cytokines previously reported.

## Main text

The GCF samples collected for the Tymkiw et al. 2011 study were used in our present study. They were from 32 subjects: 20 periodontally diseased subjects and 12 periodontally healthy subjects (Additional file 1: Figure S1) [16]. They were from 30 Caucasian, 1 Latino, and 1 Asian subjects; 40–75 years of age; and were never smokers in good general health. The periodontally diseased subjects had a diagnosis of generalized severe chronic periodontitis based on  ≥ 30% of sites with ≥ 5 mm CAL [17] and PD  ≥ 5 mm with BOP. Periodontally healthy subjects had CAL and PD ≤ 3 mm and BOP ≤ 10%. Written informed consent was obtained and GCF were collected from periodontally diseased and periodontally healthy subjects with approval from the Human Institutional Review Board, University of Iowa (IRB ID #: 201,807,763) [16]. Our present study to assess the concentrations of PD-L1 in these original samples did not meet the regulatory definition of human subject research and did not require re-review.

The GCF samples collected for the Tymkiw et al. [16] were from two healthy (PD and CAL ≤ 3 mm, BOP absent), and two diseased (PD and CAL  ≥ 5 mm with BOP) sites in each of the 20 periodontally diseased subjects. In each of the 12 periodontally healthy subjects, GCF samples were obtained from four healthy (PD and CAL ≤ 3 mm, BOP absent) sites. These samples have been stored at − 80 °C since 2011.

In this present study, PD-L1 values were determined in triplicate using a fluorescent microparticle-based immunoassay (Millipore, Billerica, MA, USA) in the Luminex 100IS (Luminex, Austin, TX, USA). PD-L1 values (pg/30 s) in each sample were interpolated from standard curves using MILLIPLEX Analyst v5.1 (Millipore, Billerica, MA, USA).

The values for CCL2, CCL3, CCL5, CCL11, CXCL8, and CXCL10 (chemokines); IL2 and IFNG (Th1 cytokines); IL3, IL4, and IL5 (Th2 cytokines); IL1A, IL1B, IL6, IL12A, IL12B, CSF2, and TNFA (pro-inflammatory cytokines); IL10 and IL13 (anti-inflammatory cytokines); and IL7 and IL15 (T-cell homeostasis) were used directly from our archived original dataset [16].

Three technically repeated PD-L1 values within each subject were averaged according to healthy or diseased status, so the analysis was based on the number of subjects rather than number of sites. A paired t-test compared PD-L1 values between healthy and diseased sites within periodontally diseased subjects (0.05 significance level) and a two sample t-test compared PD-L1 values between healthy sites of the periodontally diseased subjects and PD-L1 values from healthy sites of the periodontally healthy control subjects (0.05 significance level).

Pearson’s correlation was used to assess relationships among PD-L1, chemokine, and cytokine values from healthy sites, and correlations between PD-L1, chemokine, and cytokine values from diseased sites (0.05 significance level). No adjustments were made for multiple comparisons.

Ingenuity Pathway Analysis (Qiagen Bioinfomatics, Qiagen, Redwood City, CA, USA) was used to assess the relationships among the PD-L1, chemokine, and cytokine responses in Canonical Pathway gene ontology (GO) annotations and Function and Disease GO annotations. Fisher’s Exact Test was used to determine statistical significance.

## Results

Twenty periodontally diseased subjects contributed 53 diseased sites and 37 healthy sites; the 12 periodontally healthy control subjects had 48 healthy sites (Table 1).

**Table 1:** Ranges and means (SEM) of PD-L1 values

Status and number of subjects	Total number of healthy and diseased sites	Subjects with healthy sites	Subjects with diseased sites	Range^a^	Mean (SEM)
Periodontally diseased subjects^b^					
20	37 healthy sites	19		(0.16, 31.65)	7.49 (2.04)
	53 diseased sites		20	(0.04, 21.73)	5.44 (1.19)
Periodontally healthy control subjects					
12	48 healthy sites	12		(1.28, 11.35)	5.79 (0.92)

^a^This is the minimum PD-L1 value and maximum PD-L1 value for each particular group of interest after averaging together all healthy sites for a subject and all diseased sites for a subject

^b^One periodontally diseased subject did not have samples from healthy sites

**Fig. 1:**
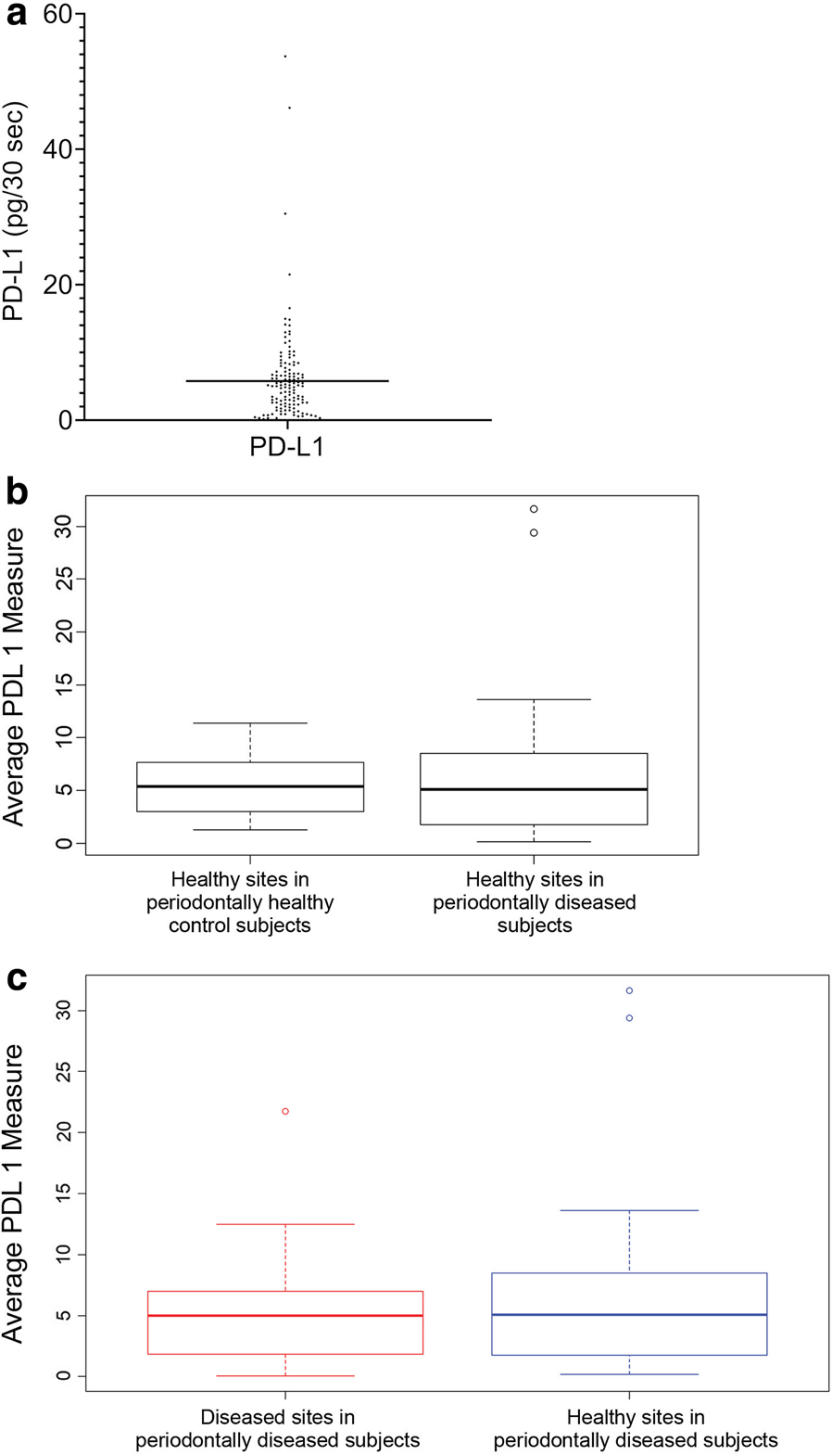
Averaged PD-L1 values from all sites in all subjects (**a**) and differences in periodontally diseased and periodontally healthy control subjects (**b, c**)

PD-L1 was detected in GCF (Fig. 1). Values in the 53 diseased sites in 20 periodontally diseased subjects ranged from 0.16 to 31.65 pg/30 s and had a mean (SEM) of 7.49 (2.04); the values of PD-L1 in 37 healthy sites in 19 periodontally diseased subjects ranged from 0.04 to 21.73 pg/30 s and had a mean (SEM) of 5.44 (1.19); and the values of PD-L1 in 48 healthy sites in 12 periodontally healthy control subjects ranged from 1.28 to 11.35 pg/30 s and had a mean (SEM) of 5.79 (0.92) (Table 1). There were no significant differences between PD-L1 values at healthy sites in the periodontally diseased subjects and PD-L1 values at healthy sites in the periodontally healthy control subjects (p = 0.46). Within the periodontally diseased subjects, there were no significant differences between PD-L1 values at healthy sites and PD-L1 values at diseased sites (p = 0.28).

PD-L1 correlated with chemokines and cytokines in healthy sites in periodontally diseased and periodontally healthy control subjects (N = 31) and in diseased sites in periodontally diseased subjects (N = 20) (Table 2). In healthy sites in periodontally diseased and periodontally healthy control subjects (N = 31), PD-L1 values were negatively correlated with IL10 (p = 0.02), CXCL8 (p = 0.001), and CCL3 (p = 0.04) values. PD-L1 values were marginally negatively correlated with IL6 values (p = 0.05). PD-L1 values were not correlated with the remaining 18 chemokines and cytokines. In diseased sites in periodontally diseased subjects (N = 20), the PD-L1 values were positively correlated with CCL11 (p = 0.02), CSF2 (p = 0.001), IFNG (p = 0.03), IL1A (p = 0.04), IL1B (p = 0.01), IL2 (p = 0.03), IL7 (p = 0.004), IL15 (p = 0.003), and CCL5 (p = 0.03) values. PD-L1 values were negatively correlated with IL12A (p = 0.01) and IL5 (p = 0.01) values. PD-L1 values were not correlated with the remaining 11 chemokines and cytokines.

**Table 2:** The correlation of PD-L1 with chemokines and cytokines in periodontally diseased and periodontally healthy control subjects

Comparison	Estimated correlation	Lower bound 95% CI	Upper bound 95% CI	p-value^a^
Healthy sites in periodontally diseased and periodontally healthy control subjects (N = 31)				
PD-L1 vs IL6	− 0.35	− 0.63	0.00	0.05
PD-L1 vs CXCL8	− 0.56	− 0.76	− 0.26	0.001
PD-L1 vs IL10	− 0.43	− 0.68	− 0.09	0.02
PD-L1 vs CCL3	− 0.37	− 0.64	− 0.01	0.04
Diseased sites in periodontally diseased subjects (N = 20)				
PD-L1 vs CCL11	0.51	0.09	0.78	0.02
PD-L1 vs CSF2	0.69	0.36	0.87	0.001
PD-L1 vs IFNG	0.49	0.06	0.77	0.03
PD-L1 vs IL12A	− 0.54	− 0.79	− 0.13	0.01
PD-L1 vs IL1A	0.46	0.03	0.75	0.04
PD-L1 vs IL1B	0.56	0.15	0.80	0.01
PD-L1 vs IL2	0.50	0.07	0.77	0.03
PD-L1 vs IRX1	− 0.55	− 0.80	− 0.15	0.01
PD-L1 vs IL7	0.61	0.23	0.83	0.004
PD-L1 vs IL15	0.64	0.27	0.84	0.003
PD-L1 vs CCL5	0.50	0.07	0.77	0.03

Taken from, the 37 healthy sites in 19 periodontally diseased subjects and the 48 healthy sites in 12 periodontally healthy control subjects

^a^Pearson’s correlation

## Discussion

Immune checkpoint co-inhibitory activity involving PD-L1 and PD-1 is thought to occur during bacterial infections, such as periodontal diseases, to protect the host from uncontrolled immune responses to pathogens and inflammation-induced tissue damage. The etiology of periodontal disease is associated with dysbiotic dental biofilms, but the host immune response to these biofilms is largely responsible for disease expression and ultimate loss of periodontal supporting tissues [18]. Periodontitis patients may display a dysregulation of their immune response in several ways including an exaggerated/inappropriate inflammatory response to the microbial challenges, unsuccessful resolutions, or autoimmune-like responses. Periodontal patients often demonstrate a reduced immune response that allows periodontopathogens to exert an exaggerated suppressive mechanism on T-cell function [8–10].

The role of PD-L1 in periodontitis has been investigated in a series of recent studies. In subjects with chronic periodontitis, elevated expression of PD-L1 was reported on leukocytes in peripheral blood and gingival lesion biopsies when compared to healthy subjects [19]. In laboratory studies, PD-L1 expression was induced on periodontal ligament cells (PDLCs) by inflammatory cytokines and periodontal pathogens [7]. In animal models of experimental periodontitis, lower values of PD-L1 on cells were associated with more severe periodontitis, while higher values of PD-L1 on cells were associated with less severe periodontitis [7]. Despite the lack of a direct correlation between periodontal tissue destruction and PD-L1 values, it is possible that higher levels of PD-L1 may induce immunosuppression limiting inflammatory tissue damage.

Previously, we compared the expression of 22 chemokines and cytokines in gingival crevicular fluid (GCF) from healthy and diseased sites subjects with periodontitis [16]. We found that periodontally diseased subjects had significantly elevated cytokine and chemokine profiles. In the present study, we assessed the presence of PD-L1 in archived GCF of these subjects and correlated values with the presence of inflammatory chemokines and cytokines in diseased and healthy sites in periodontally diseased subjects and healthy sites in periodontally healthy subjects.

PD-L1 correlated with 15 of 22 chemokine and cytokine responses. In healthy sites, PD-L1 values were negatively correlated with 4 cytokine and chemokine values; in diseased sites, PD-L1 values were positively correlated with 9 chemokine and cytokine values; and in diseased sites, PD-L1 values were negatively correlated with 2 cytokine values (Table 2). Relationships exist between PD-L1 and these same 15 chemokine and cytokine responses, which would be important in understanding their positive correlations (Table 2). PD-L1 can be induced by exposure of cells to inflammatory cytokines IL1, IL6, CSF2, IFNG, TNFA, and VEGFA [7, 20, 21]; gamma-chain cytokines IL2, IL7, IL10, IL15, and IL21 [22]; or to exposure to oral microorganisms [7] or their products [7, 23]. The direct correlation of PD-L1 with chemokine and cytokine values could be related to the ability of IL2 and CSF2 to induce the production of PD-L1 [7, 20–22]. The inverse correlation of PD-L1 with chemokine and cytokine values could be related to the ability of PD-L1 to regulate the production of IL5, IL6, CXCL8, IL12A, and CCL3.

These correlations appeared to have meaningful biological relationships. Canonical pathway gene ontology (GO) annotations for Th1 and Th2 activation contained PD-L1, IFNG, IL2, IL5, IL6, IL10, and IL12A; GO annotations for Th1 responses contained PD-L1, IFNG, IL2, IL6, IL10, and IL12A; and GO annotations for T-cell exhaustion signaling contained PD-L1, IFNG, IL6, IL10, and IL12A (Additional file 2: Figure S2).

Analysis to associate the significant responses with relevant disease functions suggested that PD-L1 had co-inhibitory activity regulating hematologic and lymphoid system development, function, and immunological disease; cellular movement and immune cell trafficking; and cell-mediated immune responses via cell-to-cell signaling and interaction (Additional file 3: Table S1). For example, PD-L1 with CSF2 and IL6 decreased leukocyte stimulation (p = 1.87E-27) and PD-L1 with IL6 decreased T lymphocyte stimulation (2.58E-22). PD-L1 with CCL11, IFNG, IL6, and IL10 decreased recruitment of leukocytes (p = 8.72E-29), PD-L1 with CSF2 and IL6 decreased induction of leukocytes (p = 1.16E-26), PD-L1 with IL6 decreased induction of lymphocytes (p = 1.33E-22). Furthermore, PD-L1 with IFNG, IL5, IL6, and IL10 decreased cellular infiltration by mononuclear leukocytes (p = 1.45E-29) and PD-L1 with IFNG, IL5, and IL10 decreased cellular infiltration by lymphocytes (p = 9.32E-28).

In future studies, we will assess the nature of PD-L1 in GCF and determine if it is a soluble protein in solution, bound on membrane fragments of dead or dying periodontal cells sloughing off in GCF, or on exomes from periodontal cells in GCF.

## Conclusion

We assessed the presence of PD-L1 in GCF from diseased and healthy sites in periodontally diseased subjects and healthy sites in periodontally healthy subjects. We then correlated the presence of PD-L1 with chemokines and cytokines. We report that (1) PD-L1 was present in GCF, (2) PD-L1 values did not vary greatly between healthy and diseased sites, and (3) PD-L1 values correlated with 15 of 22 chemokine and cytokine values.

## Limitations

Our study has several limitations:

- We used archived GCF samples from a study over 10 years ago that was designed to assess the influence of smoking on GCF cytokines in severe chronic periodontitis. The present study included only the non-smoking periodontally diseased and healthy subjects from that study. Future studies should include larger sample sizes based on power analysis using data from the present study in order to evaluate PD-L1 expression in periodontal health and disease. In addition, evaluating its expression in healthy subjects, gingivitis subject and severe periodontitis subjects would provide additional insights into PD-L1′s role in the pathogenesis of periodontitis.
- This study was cross-sectional; PD-L1 values may vary depending on periodontal disease activity. A longitudinal study would be necessary to determine the association between disease activity and PD-L1.
- The periodontal disease classification system has changed since this study was conducted, and future research should utilize criteria for health and disease categories from the 2017 World Workshop on the Classification of Periodontal and Peri-implant Diseases and Conditions [24].

## Supplementary information

**Additional file 1: Figure S1.** Gingival crevicular fluid (GCF) was collected from diseased and healthy sites in periodontally diseased subjects and healthy sites in periodontally healthy subjects. These subjects and the collection of their GCF determination of Programmed Death-Ligand 1 (PD-L1) concentrations was previously described in detail by Tymkiw et al. [20].

**Additional file 2: Figure S2. ** PD-L1 values correlated with the production of 15 chemokines and cytokines and IPA was used to show that these were meaningful biological relationships. Of these, PD-L1, IFNG, IL2, IL5, IL6, IL10, and IL12A shared common Th1 and Th2 activation; PD-L1, IFNG, IL2, IL6, IL10, and IL12A shared common Th1; and PD-L1, IFNG, IL6, IL10, and IL12A shared common T-cell exhaustion signaling canonical pathways.

**Additional file 3: Table S1**. Ingenuity Pathway Analysis (Qiagen Bioinfomatics, Qiagen, Redwood City, CA) was used to perform a Function and Disease analysis to assess the biological relevance of correlation of PD-L1 with 15 expressed chemokines and cytokines and to associate these profiles with important immune activities. The list of relevant disease functions associated with the pathway molecules was determined and ranked by category and function. Shown are the top 5–10 relevant disease functions in each category. The statistical significance was calculated within IPA using Fisher’s Exact Test.

## Data Availability

The datasets used and/or analyzed during the currently study are available from the corresponding author on reasonable request.

## Abbreviations

PD-L1: Programmed death-ligand 1 (CD274)
PD-1: Programmed death-1
GCF: Gingival crevicular fluid
Th1: T helper 1
Th2: T helper 2
CAL: Clinical attachment loss
PD: Probing depth
BOP: Bleeding on probing
GO: Gene ontology
PDLCs: Periodontal ligament cells
