# Supplementary figures and images for: PD-L1 correlates with chemokines and cytokines in gingival crevicular fluid from healthy and diseased sites in subjects with periodontitis
Source: BMC Res Notes. 2020 Nov 13;13:532. doi: 10.1186/s13104-020-05376-9 (PMC7666489; doi:10.1186/s13104-020-05376-9)

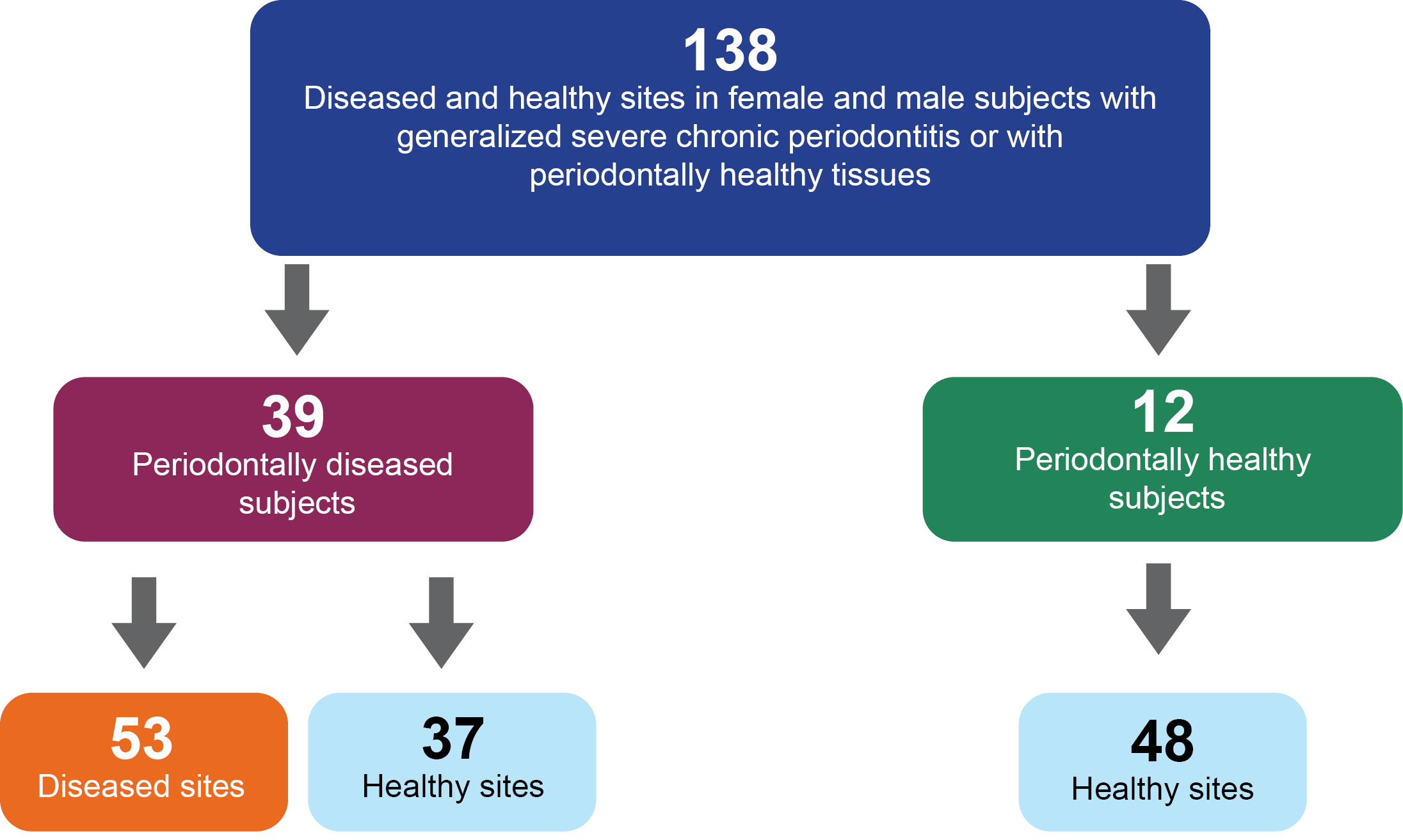

Supplement: Supplementary file 1 — Additional file 1: Figure S1. Gingival crevicular fluid (GCF) was collected from diseased and healthy sites in periodontally diseased subjects and healthy sites in periodontally healthy subjects. These subjects and the collection of their GCF determination of Programmed Death-Ligand 1 (PD-L1) concentrations was previously described in detail by Tymkiw et al. [20]. [file 13104_2020_5376_MOESM1_ESM.jpg]

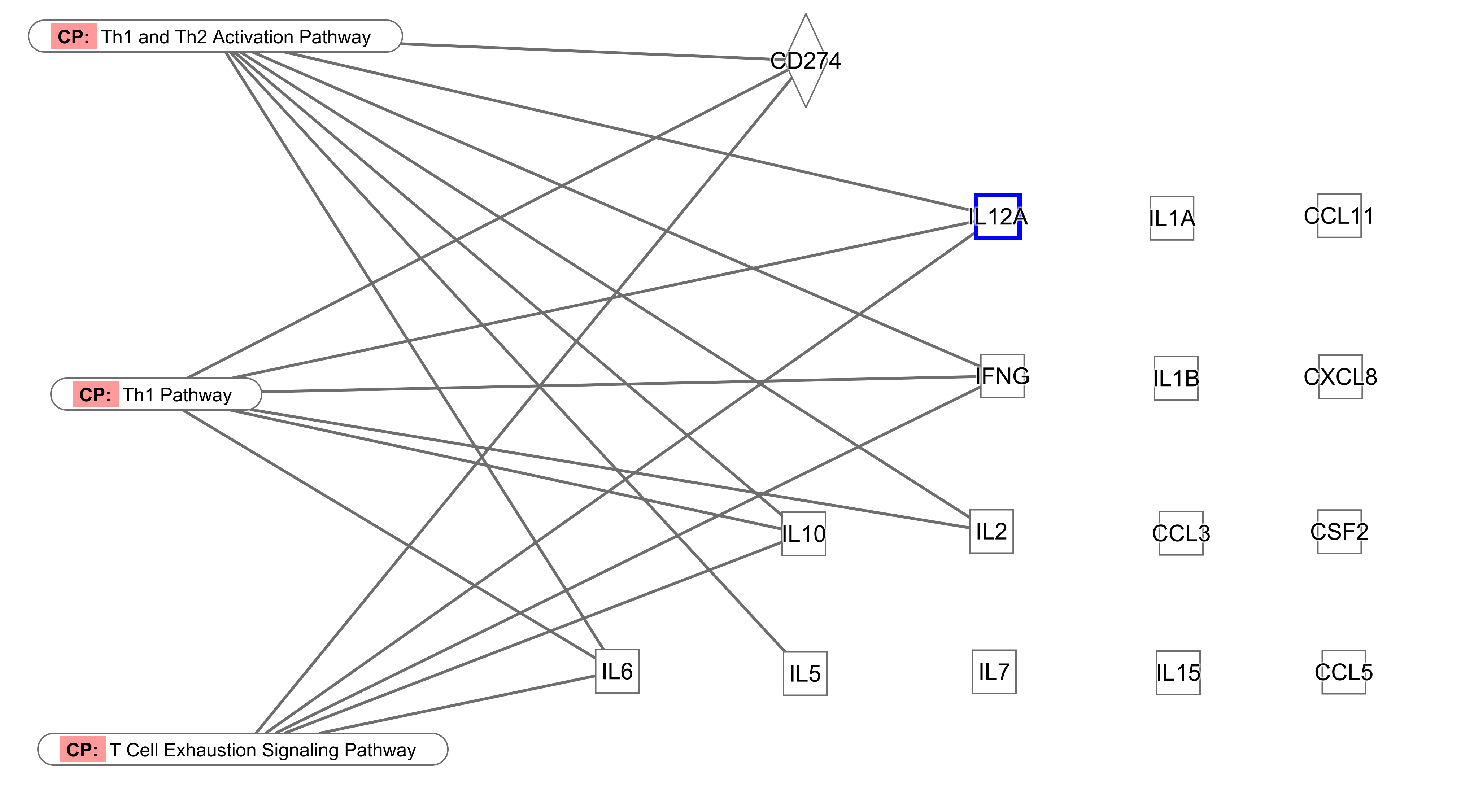

Supplement: Supplementary file 2 — Additional file 2: Figure S2. PD-L1 values correlated with the production of 15 chemokines and cytokines and IPA was used to show that these were meaningful biological relationships. Of these, PD-L1, IFNG, IL2, IL5, IL6, IL10, and IL12A shared common Th1 and Th2 activation; PD-L1, IFNG, IL2, IL6, IL10, and IL12A shared common Th1; and PD-L1, IFNG, IL6, IL10, and IL12A shared common T-cell exhaustion signaling canonical pathways. [file 13104_2020_5376_MOESM2_ESM.jpg]
