# Supplementary material for: PD-L1 correlates with chemokines and cytokines in gingival crevicular fluid from healthy and diseased sites in subjects with periodontitis
Source: BMC Res Notes. 2020 Nov 13;13:532. doi: 10.1186/s13104-020-05376-9 (PMC7666489; doi:10.1186/s13104-020-05376-9)
Supplement: Supplementary file 3 — Additional file 3: Table S1. Ingenuity Pathway Analysis (Qiagen Bioinfomatics, Qiagen, Redwood City, CA) was used to perform a Function and Disease analysis to assess the biological relevance of correlation of PD-L1 with 15 expressed chemokines and cytokines and to associate these profiles with important immune activities. The list of relevant disease functions associated with the pathway molecules was determined and ranked by category and function. Shown are the top 5–10 relevant disease functions in each category. The statistical significance was calculated within IPA using Fisher’s Exact Test. [file 13104_2020_5376_MOESM3_ESM.docx]

| **Additional file 3: Table S2.** Ingenuity Pathway Analysis (Qiagen Bioinfomatics, Qiagen, Redwood City, CA) was used to perform a Function and Disease analysis to assess the biological relevance of correlation of PD-L1 with 15 expressed chemokines and cytokines and to associate these profiles with important immune activities. The list of relevant disease functions associated with the pathway molecules was determined and ranked by category and function. Shown are the top 5-10 relevant disease functions in each category. The statistical significance was calculated within IPA using Fisher’s Exact Test. | | | |
| --- | --- | --- | --- |
| **Categories (Function and Disease analysis)** | **No. molecules** | **Molecules** | **P-value** |
| **Cell-To-Cell Signaling and Interaction, Cellular Growth and Proliferation, Hematological System Development and Function** | | | |
| Induction of cells | 13 | CD274, CSF2, CXCL8, IFNG, IL10, IL12A, IL15, IL1A, IL1B, IL2, IL5, IL6, IL7 | 1.42E-27 |
| Induction of helper T lymphocytes | 7 | CD274, IFNG, IL10, IL1B, IL2, IL5, IL6 | 1.07E-17 |
| Induction of leukocytes | 12 | CD274, CSF2, CXCL8, IFNG, IL10, IL12A, IL15, IL1B, IL2, IL5, IL6, IL7 | 1.16E-26 |
| Induction of lymphatic system cells | 11 | CD274, CSF2, IFNG, IL10, IL12A, IL15, IL1B, IL2, IL5, IL6, IL7 | 4.50E-25 |
| Induction of lymphocytes | 10 | CD274, IFNG, IL10, IL12A, IL15, IL1B, IL2, IL5, IL6, IL7 | 1.33E-22 |
| Induction of mononuclear leukocytes | 11 | CD274, CSF2, IFNG, IL10, IL12A, IL15, IL1B, IL2, IL5, IL6, IL7 | 5.02E-25 |
| Induction of T lymphocytes | 9 | CD274, IFNG, IL10, IL12A, IL1B, IL2, IL5, IL6, IL7 | 2.74E-20 |
| Stimulation of cells | 15 | CCL11, CCL5, CD274, CSF2, CXCL8, IFNG, IL10, IL12A, IL15, IL1A, IL1B, IL2, IL5, IL6, IL7 | 6.03E-26 |
| Stimulation of leukocytes | 14 | CCL11, CCL5, CD274, CSF2, CXCL8, IFNG, IL10, IL12A, IL15, IL1B, IL2, IL5, IL6, IL7 | 1.87E-27 |
| Stimulation of T lymphocytes | 11 | CD274, CSF2, IFNG, IL10, IL12A, IL15, IL1B, IL2, IL5, IL6, IL7 | 2.58E-22 |
| **Cell-To-Cell Signaling and Interaction, Cellular Movement, Hematological System Development and Function, Immune Cell Trafficking, Inflammatory Response** | | | |
| Cellular infiltration by CD4^+^ T-lymphocytes | 6 | CCL11, CCL5, CD274, IFNG, IL15, IL7 | 1.07E-13 |
| Recruitment of antigen presenting cells | 8 | CCL3, CCL5, CD274, CSF2, CXCL8, IFNG, IL1B, IL2 | 1.28E-14 |
| Recruitment of neutrophils | 11 | CCL3, CCL5, CD274, CSF2, CXCL8, IFNG, IL10, IL1A, IL1B, IL6, IL7 | 2.06E-19 |
| Recruitment of phagocytes | 12 | CCL3, CCL5, CD274, CSF2, CXCL8, IFNG, IL10, IL1A, IL1B, IL2, IL6, IL7 | 5.43E-20 |
| **Cell-To-Cell Signaling and Interaction, Hematological System Development and Function, Inflammatory Response** | | | |
| Activation of CD8^+^ T lymphocyte | 6 | CD274, IFNG, IL15, IL1A, IL2, IL6 | 8.52E-14 |
| Cell-mediated response of T lymphocytes | 9 | CD274, CSF2, CXCL8, IFNG, IL10, IL1B, IL2, IL6, IL7 | 3.39E-20 |
| Immune response of leukocytes | 13 | CCL11, CCL3, CD274, CSF2, CXCL8, IFNG, IL10, IL12A, IL15, IL1B, IL2, IL6, IL7 | 8.57E-21 |
| Th17 immune response | 6 | CD274, CXCL8, IFNG, IL10, IL1B, IL6 | 6.86E-15 |
| **Cell Cycle, Cell Death and Survival, Cellular Compromise** | | |  |
| Apoptosis of memory T lymphocytes | 5 | CD274, IFNG, IL15, IL2, IL7 | 1.23E-14 |
| Apoptosis of T lymphocytes | 12 | CCL3, CCL5, CD274, IFNG, IL10, IL12A, IL15, IL1A, IL1B, IL2, IL6, IL7 | 1.66E-19 |
| Cell division of blood cells | 7 | CD274, CSF2, IL15, IL2, IL5, IL6, IL7 | 9.96E-16 |
| Cytotoxicity of leukocytes | 13 | CCL11, CCL5, CD274, CSF2, IFNG, IL10, IL12A, IL15, IL1A, IL2, IL5, IL6, IL7 | 2.92E-26 |
| Cytotoxicity of T lymphocytes | 11 | CCL11, CCL5, CD274, IFNG, IL10, IL12A, IL15, IL1A, IL2, IL6, IL7 | 3.62E-25 |
| **Cell Morphology, Immunological Disease** | | | |
| Abnormal morphology of leukocytes | 9 | CD274, CSF2, IFNG, IL10, IL15, IL2, IL5, IL6, IL7 | 3.08E-15 |
| Morphology of leukocytes | 10 | CD274, CSF2, CXCL8, IFNG, IL10, IL15, IL2, IL5, IL6, IL7 | 2.84E-15 |
| **Cell-mediated Immune Response, Cellular Development, Cellular Function and Maintenance, Cellular Growth and Proliferation** | | | |
| Depletion of lymphocytes | 7 | CD274, CXCL8, IFNG, IL12A, IL15, IL5, IL7 | 4.84E-15 |
| Differentiation of CD4^+^ T-lymphocytes | 7 | CD274, CSF2, IL15, IL1B, IL2, IL6, IL7 | 8.66E-14 |
| Differentiation of helper T lymphocytes | 12 | CCL3, CCL5, CD274, CSF2, IFNG, IL10, IL12A, IL15, IL1B, IL2, IL6, IL7 | 1.02E-21 |
| T cell development | 13 | CCL3, CCL5, CD274, CSF2, IFNG, IL10, IL12A, IL15, IL1A, IL1B, IL2, IL6, IL7 | 1.59E-18 |
| **Cellular Development, Cellular Growth and Proliferation, Hematological System Development and Function, Lymphoid Tissue Structure and Development** | | | |
| Differentiation of leukocyte cell lines | 6 | CD274, CSF2, IL10, IL2, IL6, IL7 | 4.68E-14 |
| Differentiation of mononuclear leukocytes | 15 | CCL3, CCL5, CD274, CSF2, CXCL8, IFNG, IL10, IL12A, IL15, IL1A, IL1B, IL2, IL5, IL6, IL7 | 4.32E-21 |
| Expansion of blood cells | 10 | CD274, CSF2, IFNG, IL10, IL15, IL1A, IL1B, IL2, IL6, IL7 | 2.17E-16 |
| Expansion of T lymphocytes | 9 | CD274, CSF2, IFNG, IL10, IL15, IL1B, IL2, IL6, IL7 | 4.08E-16 |
| Function of lymphocytes | 10 | CD274, IFNG, IL10, IL12A, IL15, IL1B, IL2, IL5, IL6, IL7 | 6.99E-16 |
| NK cell proliferation | 10 | CD274, CSF2, IFNG, IL10, IL15, IL1A, IL1B, IL2, IL6, IL7 | 1.25E-19 |
| Proliferation of effector T lymphocytes | 8 | CD274, IFNG, IL10, IL15, IL1B, IL2, IL6, IL7 | 2.52E-20 |
| Proliferation of lymphatic system cells | 15 | CCL3, CCL5, CD274, CSF2, CXCL8, IFNG, IL10, IL12A, IL15, IL1A, IL1B, IL2, IL5, IL6, IL7 | 1.52E-19 |
